# Subglottic Metastatic Rectal Adenocarcinoma: A Specialist Multidisciplinary Airway Team Approach for Optimized Voice and Airway Outcome

**DOI:** 10.1155/2017/2131068

**Published:** 2017-01-05

**Authors:** Richard Heyes, Ramkishan Balakumar, Krishan Ramdoo, Taran Tatla

**Affiliations:** Department of Otolaryngology-Head and Neck Surgery, Northwick Park Hospital, London North West Healthcare NHS Trust, London, UK

## Abstract

A 56-year-old female with a background of metastatic rectal adenocarcinoma presented with a subglottic mass causing biphasic stridor. Transoral laser microsurgery and the use of fibrin glue prevented the need for tracheostomy. Six months postoperatively there was no evidence of recurrence. Laryngeal metastasis of colorectal adenocarcinoma, although remarkably rare, is perhaps more prevalent than commonly perceived and the presence of laryngeal symptoms in a patient with colorectal adenocarcinoma should raise concern. This case is presented to aid physicians should they encounter a similar presentation of metastasis to the subglottis.

## 1. Introduction

An inpatient otolaryngology consult, although often for common and benign conditions, has the potential to recognize rare disease which requires urgent surgical intervention. The presence of a mass in the larynx compromising the airway is such a circumstance. The primary presenting feature of a subglottic mass may be airway obstruction, and differentials for these masses should include primary neoplasms of the laryngeal mucosa such as adenoid cystic carcinoma or squamous cell carcinoma, laryngeal chondrosarcoma, and metastasis from a distant primary tumor. A rare case of metastasis to the subglottis causing airway compromise is presented, and its management including the novel use of a common otolaryngological material is described.

## 2. Case Report

A 56-year-old female presented with a four-week history of increasing shortness of breath, cough, and mild dysphonia. She had been treated with antibiotics for chest X-ray demonstrated pneumonia during this time but received little benefit. On examination she was found to be “wheezy” on auscultation of the chest and she was admitted under the respiratory service.

A new diagnosis of asthma was suggested for which she was started on salbutamol nebulizers and oral prednisone forty milligrams. She made little progress and suffered sporadic episodes of oxygen desaturation. On day nine of admission, a computed tomography scan of the chest demonstrated known pulmonary metastases and an irregular appearance of the larynx. On the tenth day of admission, an otolaryngology consult was sought.

The patient's medical history was significant for locally advanced, Stage IIIb (T4, N1, M0), adenocarcinoma of the rectum which was diagnosed and treated eight years prior to this episode. Adenocarcinoma treatment involved chemoradiation with Capecitabine as an adjunct for abdominoperineal excision of the rectum with vertical rectus abdominis myocutaneous flap. Four years after her initial treatment she required radio-frequency ablation for lung and liver metastases, she underwent a partial right lung resection five years after initial treatment, and six years after initial treatment she required further radio-frequency ablation of liver and lung metastases. Her metastases proved resistant to radio-frequency ablation and at the time of admission she had received four cycles of palliative Capecitabine and Mitomycin chemotherapy. She had suffered two pulmonary emboli, one six years before this episode and one six months prior, for which she took regular prophylactic low molecular weight heparin.

On otolaryngology review, she had biphasic stridor on deep breathing. Flexible nasendoscopy (FNE) was performed at the bedside which visualized a large nodular subglottic mass. Epinephrine nebulizers and intravenous dexamethasone were initiated. On the day of review, a head and neck operative list was taking place. Between cases, a specialist head and neck otolaryngologist and a specialist “difficult airway” anesthesiologist reviewed the patient with repeat FNE. A decision was quickly made to add the patient to their operative list for surgical resection of the mass while securing the airway.

**Figure 1:**
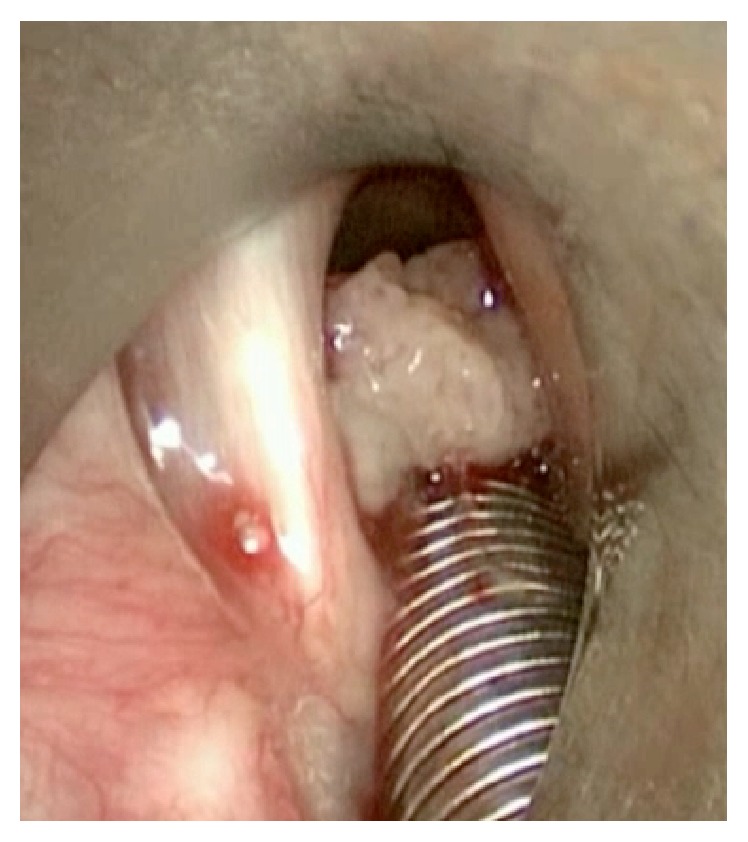
Intraoperative telescopic appearance of the subglottic tumor.

**Figure 2:**
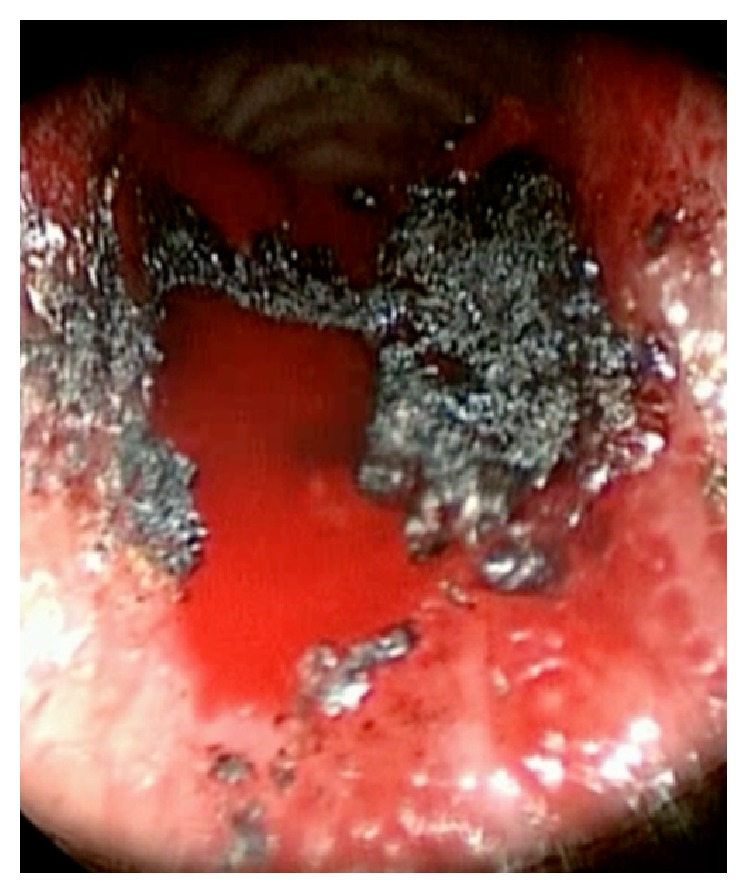
Telescopic appearance of the underlying tissues following transoral laser resection of the remnant subglottic tumor.

**Figure 3:**
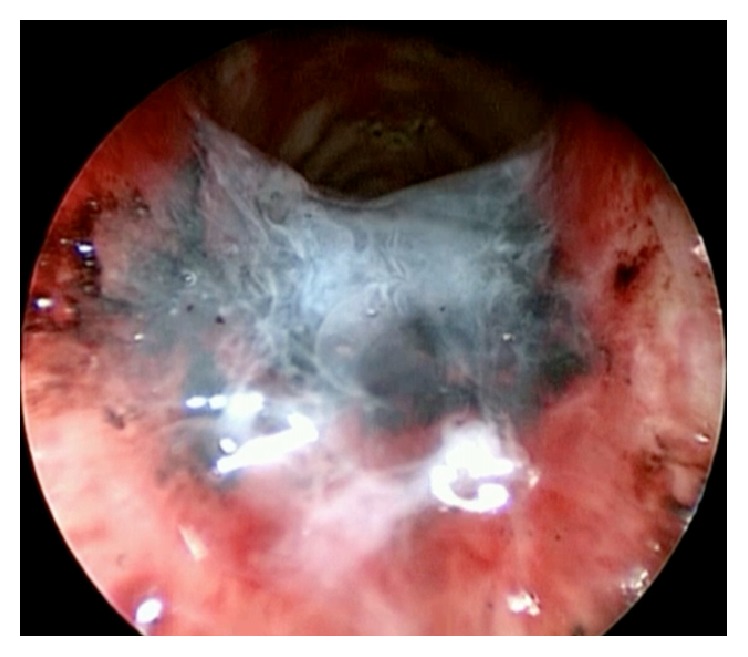
Appearance of the subglottis following the first fibrin sealant application.

Intubation was performed with the assistance of the Karl Storz C-MAC video laryngoscope and an endotracheal tube was “railroaded” beyond the subglottic mass with an Eschmann tracheal tube introducer. Microlaryngoscopy demonstrated a large sessile subglottic mass arising from the posterior commissure (Figure 1). The bulk of this mass was easily removed en masse by a laryngeal grasper. After a surgical pause to assess for bleeding, the endotracheal tube was replaced with jet ventilation to allow for laser resection of the mass remnant and its underlying mucosa; ten watts of carbon dioxide laser was used on super-pulse mode for the resection. Following laser resection the tissue was extremely friable and bleeding followed any contact (Figure 2). Therefore, in an effort to ensure no further bleeding into the airway, Tisseel fibrin glue (Baxter AG, Vienna, Austria) was applied over the site of laser ablation through a catheter inserted in the operative channel of the bronchoscope with the endoscopic applicator provided by the manufacturer. During Tisseel application, jet ventilation was held for between ninety seconds and two minutes. Two application cycles of Tisseel were employed with five minutes between applications (Figure 3). Five minutes after the second application of Tisseel cessation of anesthesia was initiated (supplementary video online in Supplementary Material available online at https://doi.org/10.1155/2017/2131068).

The mass measured twelve millimeters in its longest diameter and was histologically described as moderately differentiated adenocarcinoma morphological consistent with metastatic colorectal carcinoma, with positive expression of Cytokeratin 20 and Caudal Type Homeobox 2 without Cluster of Differentiation 7. Immunohistochemical studies for mismatch repair proteins found no evidence to support a diagnosis of Lynch syndrome. Pathological analysis is therefore strongly suggestive of primary tumor origin in the large intestine, confirming this mass as a metastasis of the patient's rectal adenocarcinoma and not a primary laryngeal tumor.

The patient was closely monitored for forty-eight hours on the high dependency unit with clear instructions to intubate if there was evidence of bleed into the airway. Subsequently she was stepped-down to the ward where significant resolution of her shortness of breath and cough were reported, and oxygen saturations above 96% were maintained throughout. Chest auscultation did not elicit any wheeze. Postoperative speech and language therapy evaluation found excellent speech and swallow function, and the patient was discharged from the speech and language therapy service prior to discharge. She was discharged from hospital one week after the procedure having had an uncomplicated postoperative course. Nine months postoperatively she was clinically stable, without evidence of tumor recurrence within the larynx.

## 3. Discussion

Metastatic disease to the larynx accounts for only 0.09 to 0.40% of all laryngeal tumors, although laryngeal metastasis may be under diagnosed since Friedmann and Osborn reported that 23.9% of patients with metastasis to the head and neck had laryngeal and tracheal diseases on autopsy [1, 2]. Ferlito et al. reported in 1988 that the most common primary sites for these metastases are the skin (melanoma) and the kidney (renal cell carcinoma); a recently published review of the English literature since 1988 proposes that colorectal adenocarcinoma is more common than previously anticipated, accounting for a quarter of 41 published cases [3, 4].

The route for hematogenous spread of colon cancer to the larynx via the systemic circulation is the inferior vena cava, right heart, lungs, left heart, aorta, external carotid artery, superior thyroid artery, and superior laryngeal artery [5]. The vertebral venous plexus has been specifically implicated in retrograde metastatic spread of colorectal carcinoma [6]. Lymphatic metastases spread via the thoracic duct, left supraclavicular lymph nodes, and subglottic lymph nodes [5]. Isolated metastasis to the larynx is extremely rare and patients with a colorectal primary are especially likely to suffer from concurrent metastasis, with lung metastases most common [7].

The location prevalence of metastatic tumors of the larynx is as follows: 39% transglottic, 27% supraglottic, 27% subglottic, and 7% glottic [4]. The site of laryngeal metastasis of colorectal adenocarcinoma is known for 14 patients: 50% were subglottic, 21% transglottic, 14% supraglottic, and 7% glottic [8]. The median age of laryngeal metastasis presentation is 59 years with a male predominance [4]. The symptoms of laryngeal metastases resemble the symptoms of primary laryngeal tumors. Common presenting complaints include dysphonia (66%) and dyspnea on exertion (60%); stridor occurs in approximately 20% of cases [4, 8]. Generally supraglottic masses are symptomatic earlier than subglottic masses, which may remain silent until airway obstruction occurs.

Although due to the rarity of laryngeal metastases treatment guidelines do not exist, symptomatic and palliative treatment is recommended in cases of concurrent distal metastasis. In isolated laryngeal metastases, or when accompanied by a single pulmonary metastasis, curative treatment should be offered following specialist multidisciplinary discussion [4, 8]. Airway protection is paramount to management. In all previous cases of rectal cancer metastasizing to the larynx, tracheostomy or extended laryngectomy has been performed. Our approach prevented significant distress to the patient (as tracheostomy recovery was her primary preoperative concern), in addition to reducing postoperative morbidity.

A successful outcome was achieved by cohesive teamwork. Early expert involvement and the presence of a specialist airway team reduced management discourse and spared the patient radical surgery. Close links with those providing intensive postoperative care and the availability of “24/7” onsite anesthesiologists and otolaryngologists allowed for safe novel intervention.

To our knowledge this is the first description of the use of fibrin glue to cover the site of laser-excised tumor in the larynx in an attempt reduce the risk of postoperative bleeding, which is associated with significant morbidity and mortality. The senior author's involvement in an unpublished case of postoperative bleeding following laser resection of a supraglottic laryngeal tumor was instrumental in the adoption of the described technique. In that case, the patient developed chest pain and ECG signs of myocardial ischemia postoperatively resulting in the administration of anticoagulative therapy and, although the wound bed was dry immediately postoperatively, basal bronchopneumonia, respiratory failure, and death resulted from presumed slow laryngeal bleeding. This experience highlights the risk of an unprotected friable wound in the airway. Fibrin glue (fibrin sealant) is composed of human plasma proteins and mimics the final pathway of the coagulation cascade, yielding a stable and insoluble clot [9]. The role of fibrin sealants throughout surgery is expanding and the use of fibrin has been described to line the repair of cricopharyngeal myotomy and in tracheal lacerations [9, 10]. Our patient suffered no complications associated with the use of fibrin glue and we have demonstrated that Tisseel can be applied to the larynx with the use of jet ventilation provided a pause in ventilation is permitted.

## 4. Conclusion

Laryngeal metastasis of colorectal adenocarcinoma, although remarkably rare, is perhaps more prevalent than commonly perceived and the presence of laryngeal symptoms in a patient with colorectal adenocarcinoma should raise concern. In this report, a specialist multidisciplinary team managed an unusual otolaryngology consult and prevented tracheostomy with the use of transoral laser microsurgery and fibrin glue. This case is presented to aid physicians should they encounter a similar presentation of metastasis to the subglottis.

## Supplementary Material

Microlaryngoscopy clearly visualized the subglottic mass. The bulk of the tumor was removed with ease. Jet ventilation allowed for laser resection of remaining tumor. The friabilty of the underlying mucosa is shown, with light touch triggering bleeding. A pause in jet ventilation permitted Tisseel application. The airway is shown shortly after the first Tisseel application demonstrating its compatibility with use in this setting.
